## [Peer Review File · Nature Communications]

REVIEWER COMMENTS

Reviewer #1 (Remarks to the Author):

The authors present an experimental work where they demonstrate the ability to trigger a lasing action in corner modes of a 2D photonic crystals. The nature of these modes is claimed to be topological, thus endowing them with a robustness to certain types of defects/perturbations. I believe this is an interesting work, well presented and technically sound. Furthermore, I believe the fields of higher-order topological phases and of topological lasing are blooming right now; this demonstration of lasing in corner modes is thus timely. However, before I can recommend this work for publication, I would like the authors to address the following concerns.

1- In a normal 2D SSH lattice, the corner modes lie at $E=0$ and are thus resonant with the middle bulk energy band. In order to spectrally resolve these states, the authors introduce a local defect at the interface (a different coupling, t_w) and a 2nd nearest-neighbour coupling. This latter perturbation is well-known to break chiral symmetry, which is the root of the topology for this kind of lattice. As a result, the energy of their corner states is directly linked to the local perturbation and to the symmetry-breaking NNN hopping. My question is the following: to which kind of perturbations these states are robust? Are they still robust to hopping energy fluctuations like in a normal SSH lattice? I doubt this, as chiral symmetry is explicitly and strongly broken. This is a particularly important point as robustness is a hallmark of topological states.

2-Regarding this question of robustness, the authors provide a qualitative study of the influence of a defect. I find this study insufficient to really understand how are their corner states protected. They only show that some corner states still exist when introducing a small perturbations relatively far from the corners. A better approach would be to show how the penetration in the bulk and/or energy of the states are affected as a function of the perturbation energy and/or spatial location (either experimentally or theoretically).

3- The authors mention that they introduce a next-nearest-neighbour coupling, which is really important to spectrally isolate the corner states. How is this controlled? Is it intrinsic or can it be tuned?

4- Is there any effect of light polarization on the observed emission spectra?

5- The title mentions multipole referring to the coupling of the corner states. I'm afraid such a title might lead to confusion, as a reader could believe the authors have implemented a multipolar topological insulator which is not the case.

6- The authors could also include a reference to a recent preprint arxiv:2002.04757, which deals with a very similar topic.

Reviewer #2 (Remarks to the Author):

In this manuscript, the authors experimentally demonstrate lasing action of corner states in two types of nanophotonic topological cavities. They demonstrate that in a small-size cavity case, corner-state lasers exhibit lower lasing threshold as compared to other types of topological lasers due to smaller mode volume and higher Q factor. In contrast to the edge states, these new topological modes can be created by controlling the coupling strength between corners. Also, as opposed to conventional photonic-crystal defect lasers, multipole corner-state lasing modes (similarly to other topological states) were shown to be immune to defects. In a larger-size cavity case, selective lasing from either edge or corner states within the topological bandgap depending on the pump position was demonstrated.

I suggest that the following comments are addressed by the authors before the manuscript is considered for publication:

1. The following recently published article is not referenced: *Light: Science & Applications*, 9, 109 (2020). It would be helpful if the results reported in the present manuscript are compared/put in context of this publication and main advantages/differences are highlighted.

2. The sentence "Four corner modes formed due to ***far-field coupling*** between the corners" may be confusing and perhaps could be modified.
3. It would be helpful if the authors could add a brief discussion of what specific applications would benefit from the unique properties of the corner-state lasers (as opposed to other types of topological lasers).
4. In Fig. 3 some discrepancies can be seen between the measured and calculated lasing modes.

Reviewer #3 (Remarks to the Author):

In the present manuscript, Kim et al. have experimentally implemented and observed lasing modes on a photonic higher-order topological insulator, the 2D SSH model. As a prototype model for corner modes, the 2D SSH model and similar variations has been widely studied and realized in many physical systems. I find this work physically sound and well written. Thus, I believe it could be suitable for Nat. Commun. if the author could consider my following comments :

1. It is stated in the paper that NNN coupling and local boundary effects break chiral symmetry, can the authors also comment on their effects on spatial symmetries since spatial symmetries are important to topological protection of higher-order modes?
2. Following the above comment, to what extent the spatial symmetries are broken during fabrication?
3. Can the authors demonstrate some unique applications/features of the high-order topological laser? I know that the topological protection is one thing but any in-gap states, being it topological or trivial, would show strong robustness to disorder.

Reply to reviewers' reports and a summary of the changes made in the revised manuscript

Response to Reviewer #1

Comment. The authors present an experimental work where they demonstrate the ability to trigger a lasing action in corner modes of a 2D photonic crystals. The nature of these modes is claimed to be topological, thus endowing them with a robustness to certain types of defects/perturbations. I believe this is an interesting work, well presented and technically sound. Furthermore, I believe the fields of higher-order topological phases and of topological lasing are blooming right now; this demonstration of lasing in corner modes is thus timely. However, before I can recommend this work for publication, I would like the authors to address the following concerns.

Our response. We thank Reviewer #1 for his/her positive evaluation of the importance of our work. We are happy to have the opportunity to address the reviewer's critical remarks, important questions, and specific suggestions.

Comment 1. In a normal 2D SSH lattice, the corner modes lie at $E=0$ and are thus resonant with the middle bulk energy band. In order to spectrally resolve these states, the authors introduce a local defect at the interface (a different coupling, t_w) and a 2nd nearest-neighbour coupling. This latter perturbation is well-known to break chiral symmetry, which is the root of the topology for this kind of lattice. As a result, the energy of their corner states is directly linked to the local perturbation and to the symmetry-breaking NNN hopping. My question is the following: to which kind of perturbations these states are robust? Are they still robust to hopping energy fluctuations like in a normal SSH lattice? I doubt this, as chiral symmetry is explicitly and strongly broken. This is a particularly important point as robustness is a hallmark of topological states.

Our response. We thank Reviewer #1 for this comment. We agree with the remark that simultaneous presence of C_{4v} and chiral symmetries in a standard 2D SSH model, with the nearest-neighbor hopping only, pins the frequency of corner states to the zero-energy level in the middle of the spectrum. In this way, corner modes appear embedded within the bulk bands of the lattice. However, to avoid mode competition in the lasing process, it is desirable to spectrally isolate corner states. Boundary effects and broken chiral symmetry modify the band structure so that corner modes fall inside the bandgap. While the existence of corner states is still guaranteed by bulk topology of the C_{4v} symmetric crystalline lattice which permits two distinct topological phases depending on the ratio of inter- and intra- cellular spacing, their resonance wavelengths and Q factors are generally susceptible to perturbations. Nevertheless, the corner-state lasing modes persist in the presence of moderate disorder and even small symmetry-breaking perturbations provided

they are weak compared to the gap size. Besides, being deeply confined, these corner states well sustain distributed bulk disorder.

We have experimentally and numerically tested robustness of the corner states by introducing a localized defect at the edge (Supplementary Fig. 5 and new Supplementary Fig. 6). In simulations, after introducing a defect even by one unit cell close to the corner, the corner modes remain within the bandgaps with negligible frequency shifts (new Supplementary Fig. 6). The corner states are also found well-defined even in the presence of displacements of holes (which emulate hopping defects in our design) and irregularities in hole sizes at the edges, up to 10% relative perturbations (new Supplementary Fig. 7).

To respond to the reviewer's comment, we have included the new Supplementary Figs. 6 and 7 in the revised Supplementary Information, and added three sentences to the revised manuscript (page 9): "In simulations, after introducing a defect even by one unit cell close to the corner, the corner modes remain within the bandgaps with negligible frequency shifts (Supplementary Fig. 6). The corner states are also found well-defined even in the presence of displacements of holes and irregularities in the sizes of holes at the edges, up to 10% relative perturbations (Supplementary Fig. 7). Besides, being deeply confined, these corner states well sustain distributed bulk disorder."

Comment 2. Regarding this question of robustness, the authors provide a qualitative study of the influence of a defect. I find this study insufficient to really understand how their corner states protected are. They only show that some corner states still exist when introducing a small perturbations relatively far from the corners. A better approach would be to show how the penetration in the bulk and/or energy of the states are affected as a function of the perturbation energy and/or spatial location (either experimentally or theoretically).

Our response. Following the reviewer's recommendation, we have additionally studied the frequency stability of corner states against position of a defect at the edge. Three cases chosen for illustration are presented in the new Supplementary Fig. 6. The resonant wavelengths and Q factors of corner modes were calculated numerically in full-wave modeling. Our numerical simulations verify sustainability of corner states revealing negligible frequency shifts even for a defect placed by one lattice constant close to the corner. The imperfection of the structure mainly affects Q factors. However, all four multipolar modes remain well-defined with their high- Q modal profiles only locally perturbed.

To respond to the reviewer's comment, we added the new Supplementary Fig. 6 to the revised Supplementary Information.

[New Supplementary Fig. 6]

Comment 3. The authors mention that they introduce a next-nearest-neighbour coupling, which is really important to spectrally isolate the corner states. How is this controlled? Is it intrinsic or can it be tuned?

Our response. Next-nearest-neighbour coupling is typically present in electromagnetic resonant structured materials, such as particle arrays, metasurfaces, and photonic crystal slabs. For our sample, we did not perform any subtle fine-tuning. The strength of such long-range coupling can be altered by changing parameters of the structure, such as a refractive index, sizes of holes, and lattice constant.

We have added a sentence to the Methods in the revised manuscript: “The strength of the long-range electromagnetic coupling in real resonant metasurfaces can be tuned by changing the structural parameters.”

Comment 4. Is there any effect of light polarization on the observed emission spectra?

Our response. The effect of light polarization on the observed emission spectra depends on the corner modes. For example, there is little polarization effect in the quadrupole and monopole modes, whereas the dipole modes exhibit the preferred polarization directions.

We have included the measured polarization data in the insets of Figs. 3b, e, h, and k. In addition, we have added a sentence to the revised manuscript (page 7): “**Fourth, there is little polarization effect in the quadrupole and monopole modes, whereas the dipole modes exhibit the preferred polarization directions (Figs. 3b, 3e, 3h, 3k; insets).**” The figure caption is as follows: “**Insets, measured output intensity of each mode as a function of the polarization angle using a linear polarizer in front of the spectrometer. 0° and 90° are the horizontal and vertical directions, respectively.**”

[New Fig. 3b, e, h, k (insets)]

Comment 5. The title mentions multipole referring to the coupling of the corner states. I'm afraid such a title might lead to confusion, as a reader could believe the authors have implemented a multipolar topological insulator which is not the case.

Our response. We agree with this remark. Following the reviewer's advice, we have changed the title as follows: “**Multipolar lasing modes from topological corner states**”.

Comment 6. The authors could also include a reference to a recent preprint arXiv: 2002.04757, which deals with a very similar topic.

Our response. As the reviewer suggested, we have included this paper into the reference list: “**35. Han, C., Kang, M. & Jeon, H. Lasing at multi-dimensional topological states in a two-dimensional photonic crystal structure. *ACS Photonics*, online publication (2020)**”.

Response to Reviewer #2

Comment. In this manuscript, the authors experimentally demonstrate lasing action of corner states in two types of nanophotonic topological cavities. The demonstrate that in a small-size cavity case, corner-state lasers exhibit lower lasing threshold as compared to other types of topological lasers due to smaller mode volume and higher Q factor. In contrast to the edge states, these new topological modes can be created by controlling the coupling strength between corners. Also, as opposed to conventional photonic-crystal defect lasers, multipole corner-state lasing modes (similarly to other topological states) were shown to be immune to defects. In a larger-size cavity case, selective lasing from either edge or corner states within the topological bandgap depending on the pump position was demonstrated.

I suggest that the following comments are addressed by the authors before the manuscript is considered for publication:

Our response. We thank Reviewer #2 for his/her positive evaluation of the importance of our work. We are happy to have the opportunity to address the reviewer's critical remarks, important questions, and specific suggestions.

Comment 1. The following recently published article is not references *Light: Science & Applications*, 9, 109 (2020). It would be helpful if the results reported in the present manuscript are compared/put in context of this publication and main advantages/differences are highlighted.

Our response. We agree with the reviewer that this recent paper is an excellent piece of work showing the low-threshold topological nanolaser in a single corner, and have happily cited it in our revised manuscript: “34. Zhang, W., Xie, X., Hao, H., Dang, J., Xiao, S., Shi, S., Ni, H., Niu, Z., Wang, C., Jin, K., Zhang, X. & Xu, X. Low-threshold topological nanolasers based on the second-order corner state. *Light: Science & Applications* 9, 109 (2020)”. However, our work, its motivation and implications have significant differences from the focus and experimental approach of that paper. We theoretically substantiated the existence of *multipolar corner modes* originating from coupling between corner states, and experimentally demonstrated and identified *all of the four corner-state lasers* using our *hyperspectral imaging system*. In particular, dipole corner-state lasers with a diagonal antinode intensity was observed for the first time. Furthermore, we observed corner- and edge-state lasing modes separately by reducing coupling between the corners in a large-size cavity. Our experimental findings conclusively link our work with non-Hermitian physics and multipolar electrodynamics exploiting collective resonances.

To emphasize this point, we have added several sentences to the revised the manuscript, as follows (page 10):

“Very recently, lasing in a single corner has been reported for similar nanopatterned structures^{34,35}. Those corner lasers, however, operate at low temperatures³⁴ or do not show the unique properties of multipolar lasing in coupled corner modes with different field profiles and Q factors^{34,35}. In this work we have conclusively demonstrated the stable multipole corner modes with antinode intensities at two or four corners as a result of coupling between the corners. In particular, dipole corner-state lasers with a diagonal antinode intensity have been observed for the first time to our knowledge. Remarkably, the coupling between the corners has been shown to be controlled and adjusted on purpose by gain/loss contrast distribution over the PTI cavity.”

Comment 2. The sentence “Four corner modes formed due to ***far-field coupling*** between the corners” may be confusing and perhaps could be modified.

Our response. We agree with the reviewer. The sentence was revised as follows (page 10): “Four corner modes formed due to the coupling between the corners”.

Comment 3. It would be helpful if the authors could add a brief discussion of what specific applications would benefit from the unique properties of the corner-state lasers (as opposed to other types of topological lasers).

Our response. Compared to edge-state topological lasers, we find that the corner-state lasers exhibit lower lasing threshold due to smaller mode volume and high Q factor. They also have an advantage of being stable to the distributed bulk disorder by contrast to the recently demonstrated bulk-state topological laser (Ref. [32]). Therefore, corner-state nanocavities appear feasible for design of low-power nanoscale lasers and sensors. They can be readily integrated as compact active elements into the nanophotonic platform to create a fully functional topological circuitry not limited to waveguides and edge-state operation.

To emphasise this aspect, we have added a sentence to the Discussion section as follows (page 11): “Furthermore, combining topological properties and practicability of conventional photonic-crystal defect lasers, corner-state nanocavities can find applications in the implementation of nanoscale lasers with improved reliability, controllability and low lasing threshold.”

Comment 4. In Fig. 3 some discrepancies can be seen between the measured and calculated lasing modes.

Our response. For a more accurate comparison between the measured and calculated lasing modes in Fig. 3a, d, g, j, we changed the calculated ones from $|E|^2$ profiles to the

z-components of the Poynting vectors. The calculation of the z-component of the Poynting vector was performed at a z-position of 300 nm above the slab. The asymmetric mode profiles of the measured images originate from the slightly different coupling strengths between the corners due to the fabrication imperfection, as we discussed in the manuscript (page 8).

We have revised Fig. 3a, d, g, j (right images) and added two sentences to the figure caption: “calculated z-components of the Poynting vectors (right). The calculation is performed at a z-position of 300 nm above the slab.”

[New Fig. 3a, d, g, j (right images)]

Response to Reviewer #3

Comment. In the present manuscript, Kim et al. have experimentally implemented and observed lasing modes on a photonic higher-order topological insulator, the 2D SSH model. As a prototype model for corner modes, the 2D SSH model and similar variations has been widely studied and realized in many physical systems. I find this work physically sound and well written. Thus, I believe it could be suitable for Nat. Commun. if the author could consider my following comments:

Our response. We thank Reviewer #3 for the favourable evaluation of our work. We are happy to have the opportunity to address the reviewer's critical remarks, important questions, and specific suggestions.

Comment 1. It is stated in the paper that NNN coupling and local boundary effects break chiral symmetry, can the authors also comment on their effects on spatial symmetries since spatial symmetries are important to topological protection of higher-order modes?

Our response. To address this question, we have provided symmetry analysis of the model Hamiltonian with NNN coupling in Supplementary Information (new Supplementary Note 1). Specifically, we show that NNN interactions do not violate C_{4v} symmetry of the lattice. The boundary effects we introduced only alter the edge- and corner-state frequencies.

We have added a sentence to the Methods in the revised manuscript: “**Importantly, NNN hopping in our model does not violate C_{4v} symmetry of the lattice, and the boundary effects only alter the edge- and corner-state frequencies (see Supplementary Note 1).**”

Comment 2. Following the above comment, to what extent the spatial symmetries are broken during fabrication?

Our response. We can estimate how broken the spatial symmetries are during fabrication, using the split wavelength (~ 3.8 nm) of the degenerate dipole corner-state modes. Since $\Delta\lambda/\lambda$ is 0.25% ($\Delta\lambda$: split wavelength, λ : wavelength of the dipole mode), it can be thought that the spatial symmetries are 0.25% broken.

To respond to the reviewer's comment, we have added a sentence to the revised manuscript as follows (page 8): “**It can be thought that the spatial symmetries are only 0.25% broken during fabrication, if we consider the small split wavelength of the degenerate dipole corner-state modes.**”

Comment 3. Can the authors demonstrate some unique applications/features of the high-order topological laser? I know that the topological protection is one thing but any in-gap states, being it topological or trivial, would show strong robustness to disorder.

Our response. We believe that sustainability to imperfections is the important feature which in our work was experimentally demonstrated for distinct multipolar corner-state lasing modes for the first time to our knowledge (Supplementary Fig. 5). We note that, compared e.g. to the in-gap edge-state topological lasers, our corner-state lasers exhibit lower lasing threshold due to smaller mode volume and higher Q factors (second paragraph on page 8). In a broader scope, the concepts of band topology suggest *systematic approaches* for smart design of light-emitting devices, engineering both near-field distributions and emission profiles, while conventional photonic-crystal defect lasers typically require fine-tuning of parameters and careful band-gap engineering.

To highlight this point, we have added a sentence to the Discussion section as follows (page 11): “Furthermore, combining topological properties and practicability of conventional photonic-crystal defect lasers, corner-state nanocavities can find applications in the implementation of nanoscale lasers with improved reliability, controllability and low lasing threshold.”

REVIEWER COMMENTS

Reviewer #1 (Remarks to the Author):

I have read the reponse of the authors carefully, and I must admit that I am only half-convinced by their arguments concerning the protection of their topological states (my previous point 1). The authors mention that: "In simulations, after introducing a defect even by one unit cell close to the corner, the corner modes remain within the bandgaps with negligible frequency shifts (new Supplementary Fig. 6). The corner states are also found well defined even in the presence of displacements of holes (which emulate hopping defects in our design) and irregularities in hole sizes at the edges, up to 10% relative perturbations (new Supplementary Fig. 7)."

I find this argument insufficient, as it is a very qualitative statement that could apply similarly to any gap states sufficiently far from the bands. The real question is rather: is there any property that is completely robust to the presence of certain types of perturbations. This is the real hallmark of topology, and it is the case, e.g., in the normal ssh model where the energy of edge states is completely unaffected by perturbations in the coupling strengths.

I do not think this type of real topological protection is present here, and I would like the authors to either state it very clearly (such that the reader is not misled to think that these states are remarkably more robust than trivial edge states) or clearly prove that such a topological protection still exists in their system. In this sense, providing a comparison between the influence of a defect on topological and non-topological states (both spectrally localized far from the bands) could be very convenient to best appreciate the topological nature of their edge states.

I think this is a crucial point that needs to be addressed in papers dealing with topological protection. If this point is addressed convincingly and without concerns, I do not need to see the manuscript again and would recommend it for publication. Otherwise, I am open to participate in a third round of review .

A very minor point: I find Fig. 7 of the Suppl. very difficult to read. I would suggest presenting a normal 1D graph for only one perturbation at a time .

Reviewer #2 (Remarks to the Author):

The authors addressed all my questions, so I recommend publication .

Reviewer #3 (Remarks to the Author):

In the reply, the author has satisfactorily addressed all my comments and made proper changes to both main text and supplementary materials. I believe, now , the manuscript should be published in Nat. Commun. with its current form.

Reply to reviewers' reports and a summary of the changes made in the revised manuscript

Reviewer #1

I have read the response of the authors carefully, and I must admit that I am only half-convinced by their arguments concerning the protection of their topological states (my previous point 1). The authors mention that: "In simulations, after introducing a defect even by one-unit cell close to the corner, the corner modes remain within the bandgaps with negligible frequency shifts (new Supplementary Fig. 6). The corner states are also found well defined even in the presence of displacements of holes (which emulate hopping defects in our design) and irregularities in hole sizes at the edges, up to 10% relative perturbations (new Supplementary Fig. 7)."

I find this argument insufficient, as it is a very qualitative statement that could apply similarly to any gap states sufficiently far from the bands. The real question is rather: is there any property that is completely robust to the presence of certain types of perturbations. This is the real hallmark of topology, and it is the case, e.g., in the normal ssh model where the energy of edge states is completely unaffected by perturbations in the coupling strengths.

I do not think this type of real topological protection is present here, and I would like the authors to either state it very clearly (such that the reader is not misled to think that these states are remarkably more robust than trivial edge states) or clearly prove that such a topological protection still exists in their system. In this sense, providing a comparison between the influence of a defect on topological and non-topological states (both spectrally localized far from the bands) could be very convenient to best appreciate the topological nature of their edge states.

I think this is a crucial point that needs to be addressed in papers dealing with topological protection. If this point is addressed convincingly and without concerns, I do not need to see the manuscript again and would recommend it for publication. Otherwise, I am open to participate in a third round of review.

Our response

In our previous revision we have provided a response to this question and included numerical results demonstrating practical robustness of the corner states when localized defects are introduced at the edge (Figs. 6 and 7 in Supplementary Information). Such simulations are typically performed to test stability of topological states, in particular for the systems fabricated by using actual semiconductor materials, as discussed in the references [14], [15], [20], [21], [22], and also [R1]: Second-order topology and multidimensional topological transitions in sonic crystals. *Nature Physics* 15, 582 (2019).

To address the additional question of Reviewer #1 about robustness of the corner states in our system, we have obtained new results and now provide a direct comparison between the influence of a bulk defect on topological states (corner-state modes) and

non-topological states (defect-cavity modes) when both are spectrally localized far from the bands, as was suggested by Reviewer #1. As shown in Fig. R1, the frequencies of the defect-cavity modes are sensitive to a bulk disorder while the frequencies of the corner states are robust. We reproduce this figure below.

[Fig. R1] Frequency differences from their original values for the corner-state (red) and defect-cavity modes (black), as a function a bulk disorder.

To respond to the reviewer’s comment, we have modified our discussions in the main manuscript and have expanded Supplementary Information including new Fig. R1 (as new Fig. 8). Also, we have added a new reference to the revised reference list (Ref. [34]).

In addition, we clarified in the previous revision that NNN hopping in our model does not violate C_{4v} symmetry of the lattice, while it breaks the chiral symmetry inevitably (see Supplementary Note 1). However, the corner states are still observed being stipulated by the nontrivial dipolar polarization, conceptually related to the crystalline topological phase (see e.g. Ref. [17]). We have extended discussions in Supplementary Information (Supplementary Note 1, section 2) which support the improved stability of corners states in our structure against the conventional defect modes.

Reviewer #1

A very minor point: I find Fig. 7 of the Suppl. very difficult to read. I would suggest presenting a normal 1D graph for only one perturbation at a time.

Our response

We have revised Fig. 7 in Supplementary Information, as was suggested by Reviewer #1. For presenting our results better, we added normal 1D graphs with fixed Δd_2 and Δr_2 as Supplementary Figs. 7c and 7f.

Reviewer #2

The authors addressed all my questions, so I recommend publication.

Our response

We thank Reviewer #2 for his/her positive evaluation of our work, and the explicit recommendation for acceptance.

Reviewer #3

In the reply, the author has satisfactorily addressed all my comments and made proper changes to both main text and supplementary materials. I believe, now, the manuscript should be published in Nat. Commun. with its current form.

Our response

We thank Reviewer #3 for his/her positive evaluation of our work, and the explicit recommendation for acceptance.

REVIEWERS' COMMENTS

Reviewer #1 (Remarks to the Author):

I am happy with the modifications provided by the authors. I believe they improved the manuscript. I now recommend its publication.

**Reply to reviewers' reports
and a summary of the changes made in the revised manuscript**

Reviewer #1

I am happy with the modifications provided by the authors. I believe they improved the manuscript. I now recommend its publication.

Our response

We thank Reviewer #1 for his/her positive evaluation of our work and the explicit recommendation for acceptance.