# Supplementary material for: Multipolar lasing modes from topological corner states
Source: Nat Commun. 2020 Nov 13;11:5758. doi: 10.1038/s41467-020-19609-9 (PMC7666194; doi:10.1038/s41467-020-19609-9)
Supplement: Supplementary file 1 — Supplementary Information [file 41467_2020_19609_MOESM1_ESM.pdf]

Supplementary Information

Multipolar lasing modes from topological corner states

Kim *et al.*

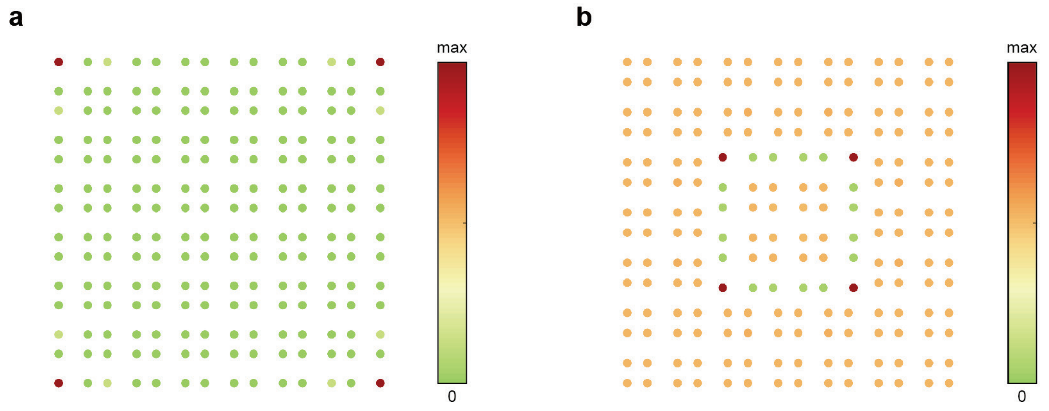

Supplementary Figure 1. Zero-energy corner states in 2D SSH lattice. a, Intensity plot of corner states in a finite lattice of expanded quadrumers at $\omega = 0$ (coupling strengths $t_a = 3.3$ and $t_b = 1$). **b,** Charge probability density of the zero-energy band calculated for a square-shaped domain wall with a weak interface coupling (coupling strengths $t_a = 6$ and $t_b = 1$). Sharp peaks at four corners sit on the average density of the bulk.

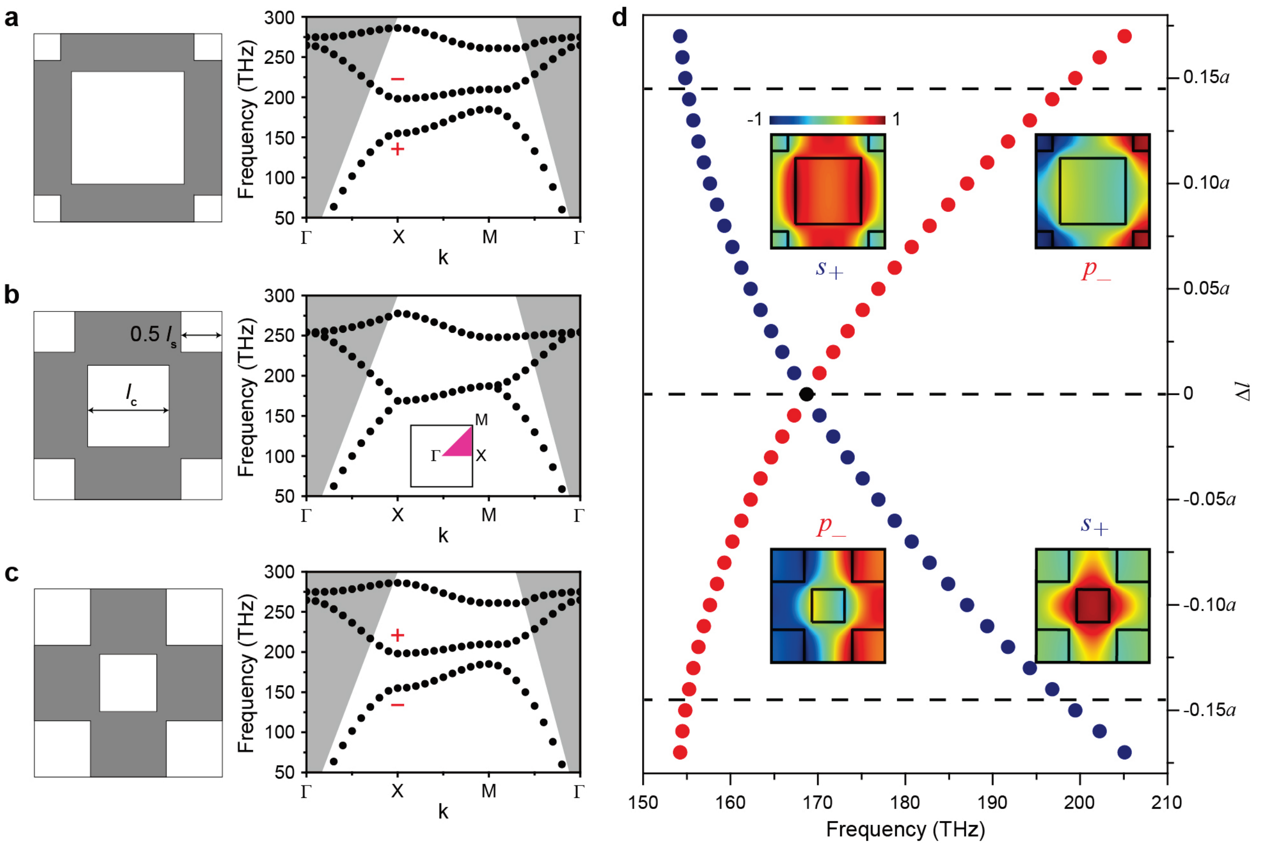

Supplementary Figure 2. Photonic band structures and topological phase transition. a-c, Schematics (left) and corresponding photonic band structures (right) of topologically trivial (**a**), symmetric (**b**), and topologically nontrivial (**c**) unit cells. The structural parameters of Figure 1d-e are used: (l_c, l_s) = (0.58a, 0.29a) (**a**), (0.435a, 0.435a) (**b**), and (0.29a, 0.58a) (**c**). The gray regions in the band structures indicate the light cone. **d,** Calculated frequency at X point of a reciprocal lattice as a function of $\Delta l = l_c - 0.435a$. Topological phase transition occurs with changing lattice structure. Inset, normalized magnetic-field (H_z) profiles in the topologically trivial (upper) and nontrivial (lower) unit cells.

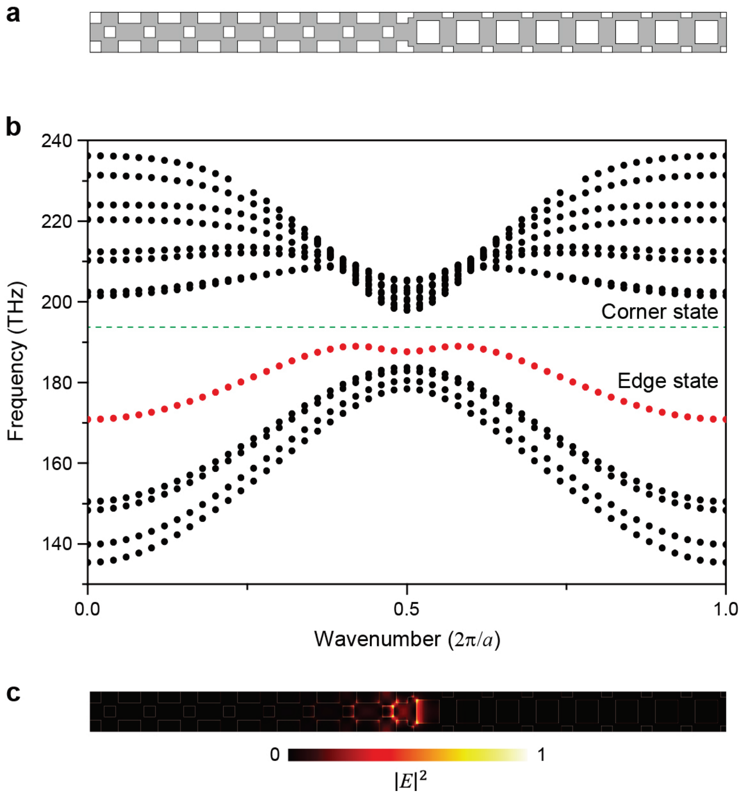

Supplementary Figure 3. Projected band structure of the edge state at the 1D PTI interface. **a,** Schematic of the 1D PTI interface. **b,** Calculated photonic band structure of the 1D PTI interface. The edge state (red) and corner state (green) are shown in the bulk bandgap. **c,** Normalized electric-field intensity ($|\mathbf{E}|^2$) profile of the edge state.

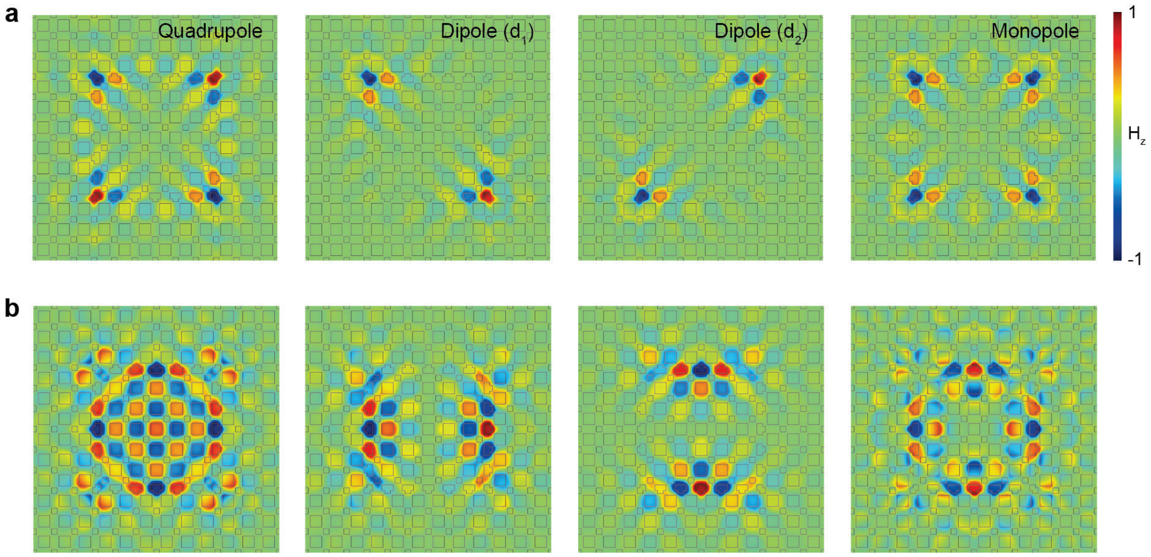

Supplementary Figure 4. Field profiles of corner and edge states. Calculated magnetic-field (H_z) profiles of (**a**) corner-state and (**b**) edge-state modes in a PTI cavity with 6 × 6 topological unit cells with a = 500 nm, l_1 = 0.58 a, l_2 = 0.29 a, and h = 275 nm, where a is the lattice constant and h is the slab thickness. These structural parameters are the same as those in Figure 1d-e. The calculated frequencies are (**a**) 194.4, 193.7, 193.7, and 193.0 THz (from the left to right) and (**b**) 188.8, 187.7, 187.7, and 187.2 THz (from the left to right). In **a**, the corner-state modes are the quadrupole, dipole (d_1), dipole (d_2), and monopole modes (from the left to right), and their calculated Q factors are 4.0×10^4, 3.6×10^4, 3.6×10^4, and 2.6×10^4, respectively. For comparison, we calculate the uncoupled corner-state mode: its frequency is 193.6 THz and Q factor is 1.8×10^4, when the distance between the corner and simulation boundary is 12a.

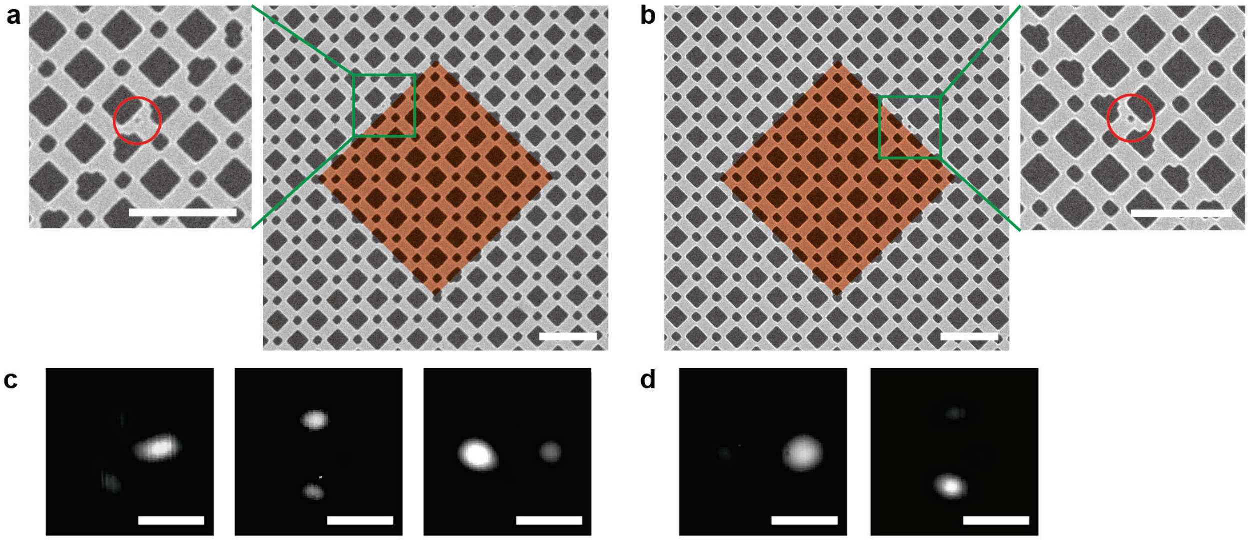

Supplementary Figure 5. Experimental robustness of topological corner-state lasers. Measurement of multipole corner-state lasing modes with a defect (square hole) introduced at the interface between the trivial and nontrivial domains. **a** and **b,** SEM images of the fabricated PTI cavities. The red-color regions (false color) indicate the nontrivial domains. Insets, magnified SEM images. The red circles in the insets indicate the fabricated defects. All scale bars, 1 μm. **c,** Measured lasing mode images in **a**. Quadrupole and two dipole modes are observed with wavelengths of 1506.2 nm, 1509.9 nm, and 1513.8 nm, respectively (from the left to right). Scale bars, 5 μm. **d,** Measured lasing mode images in **b**. Two dipole modes are observed with wavelengths of 1505.2 nm and 1507.4 nm, respectively (from the left to right). Scale bars, 5 μm.

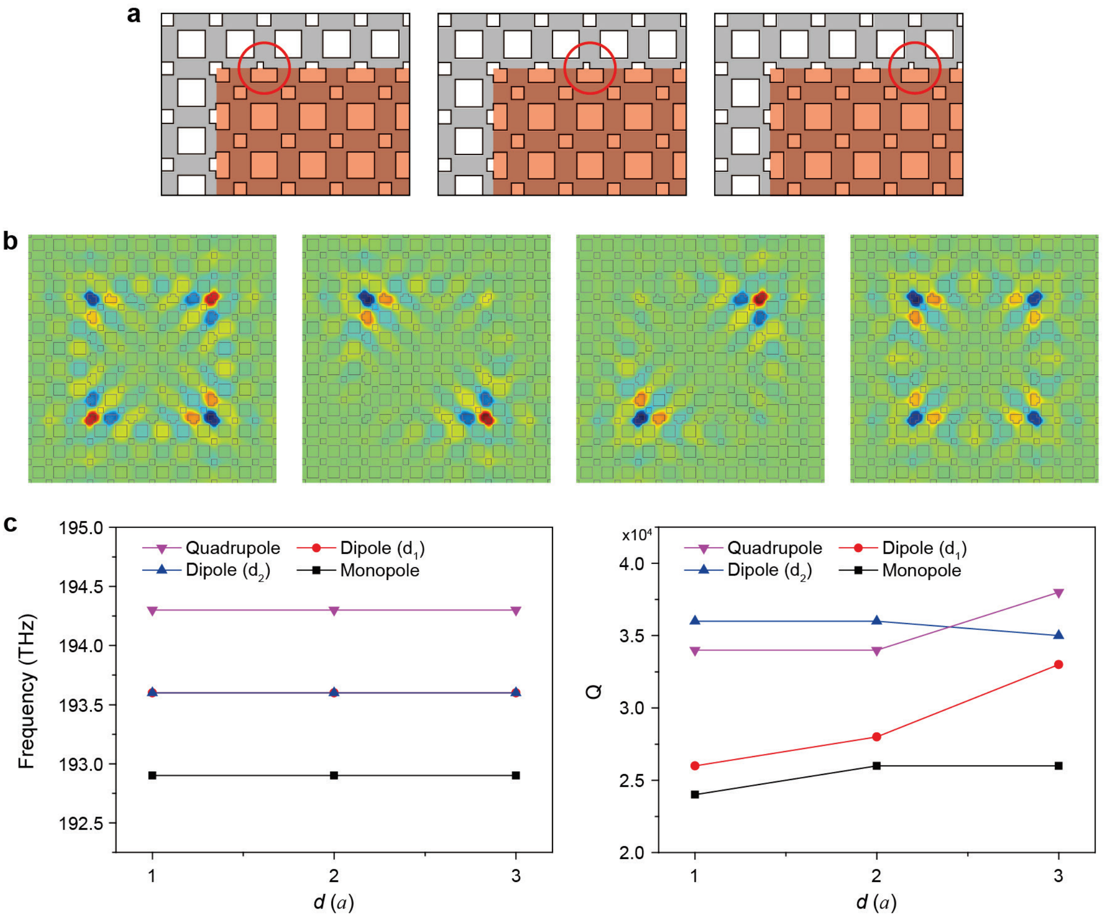

Supplementary Figure 6. Numerical simulations for robustness against a defect. FEM simulation is performed to study the frequency stability of corner states against position of a defect at the edge. **a,** Three cases chosen for illustration. The red circle is the defect and the red-color region is the nontrivial domain. The distance between the defect and corner, *d*, varies from *a* to 3*a* (left to right), where *a* is the lattice constant (500 nm). Other structural parameters are the same as those in Supplementary Figure 4. **b,** Calculated magnetic-field (H_z) profiles of the corner-state modes, quadrupole, dipole (d_1), dipole (d_2), and monopole (left to right) at $d = a$. **c,** Calculated resonant frequencies (left) and *Q* factors (right) of corner modes as a function of *d*. The numerical simulations verify sustainability of corner states revealing negligible frequency shifts even for a defect placed by one lattice constant close to the corner. Although the imperfection of the structure mainly affects *Q* factors, all four multipolar modes remain well-defined with their high-*Q* modal profiles only locally perturbed.

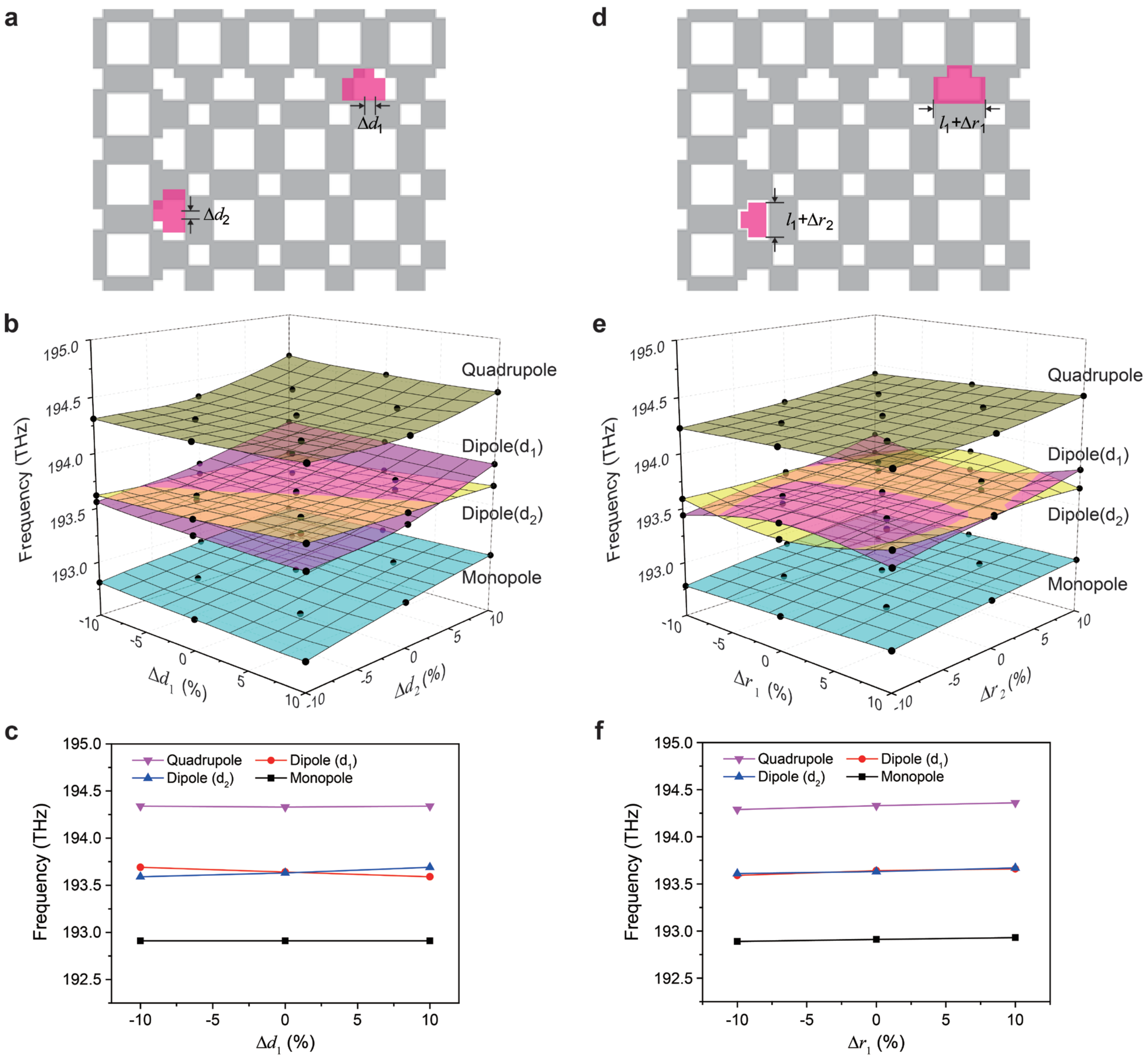

Supplementary Figure 7. Numerical simulations for robustness against displacements and irregularities. FEM simulations are performed to study the frequency stability of corner states against displacements of holes (**a-c**) and irregularities in the sizes of holes at the edges (**d-f**). **a-c,** The PTI structure with displacements of holes at the top edge (Δd_1) and left edge (Δd_2) (**a**); calculated frequencies of corner modes as functions of Δd_1 and Δd_2 (**b**); 1D graph at $\Delta d_2 = 0$ (**c**). **d-f,** The PTI structure with irregularities in the sizes of holes at the top edge (Δr_1) and left edge (Δr_2) (**d**); calculated frequencies of corner modes as functions of Δr_1 and Δr_2 (**e**); 1D graph at $\Delta r_2 = 0$ (**f**). The corner states are found well-defined even in the presence of displacements of holes and irregularities in the sizes of holes at the edges, up to 10% relative perturbations.

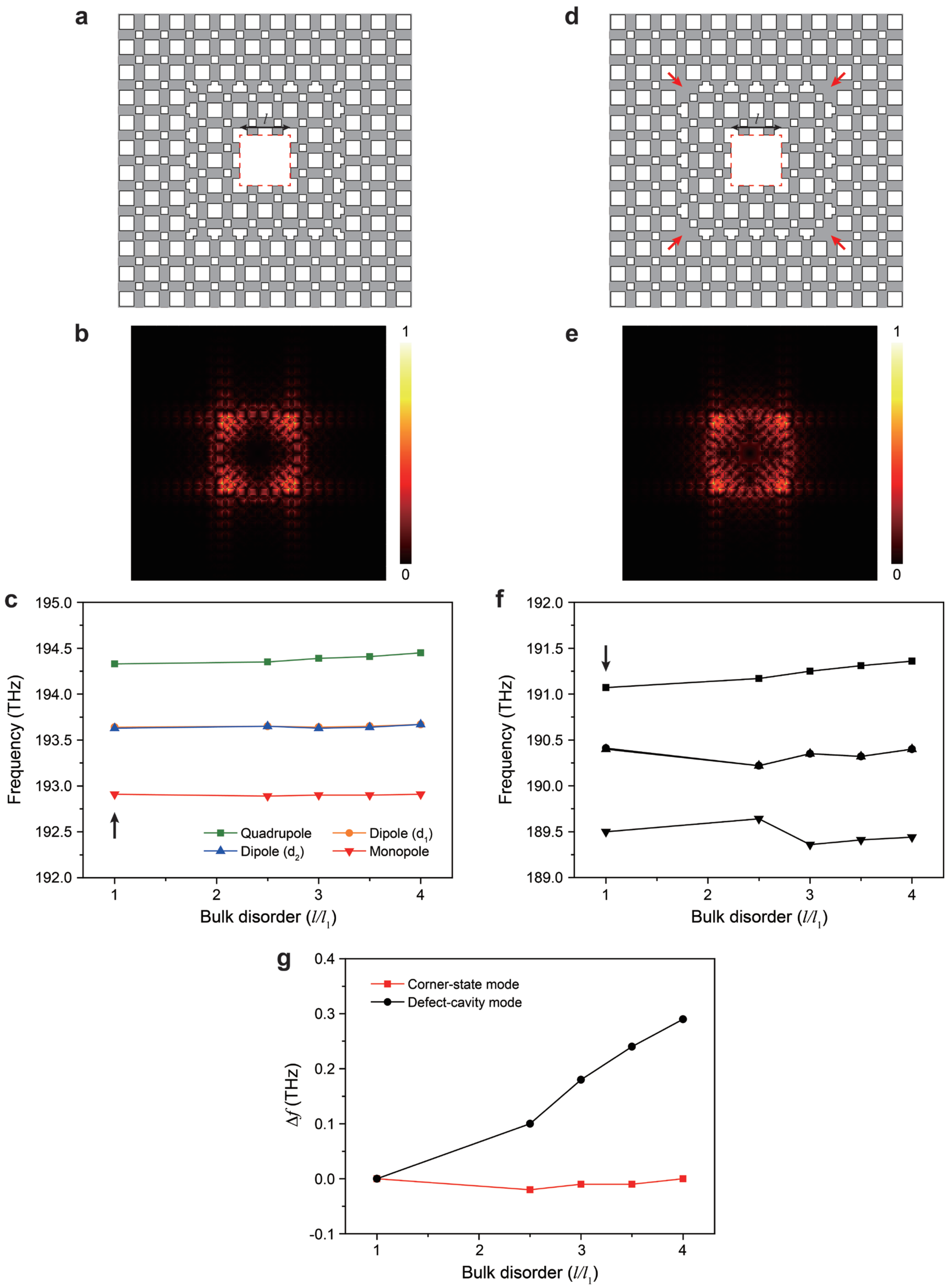

Supplementary Figure 8. Influence of a bulk disorder on corner states and defect-cavity states. a, Schematic of the PTI cavity with a bulk disorder. The size of the square air hole at the

center, l, is changed from l_1 (original value) to $4l_1$, where $l_1 = 0.58a$ and $a = 500$ nm. **b-c,** Calculated results in **a**: |**E**| profile of the monopole corner mode at $l = 3.5l_1$ (**b**) and resonant frequencies of corner-state modes as a function of bulk disorder (l/l_1) (**c**). **d,** Schematic of the defect cavity with a bulk disorder. Four air holes at the corner positions (red arrows) are removed to excite defect-cavity modes. The same bulk disorder as **a** is introduced at the center. **e-f,** Calculated results in **d**: |**E**| profile of a defect-cavity mode at $l = 3.5l_1$ (**e**) and resonant frequencies of defect-cavity modes as a function of bulk disorder (l/l_1) (**f**). **g,** Frequency difference from the original value, Δf, as a function of bulk disorder (l/l_1), for the corner-state mode of **b** (red) and the defect-cavity mode of **e** (black). The frequencies corresponding to these modes are indicated by the arrows in **c** and **f**. Both modes are spectrally localized far from the bands. We note that the frequency of the defect-cavity mode is sensitive to a bulk disorder, while the frequency of the corner state is robust.

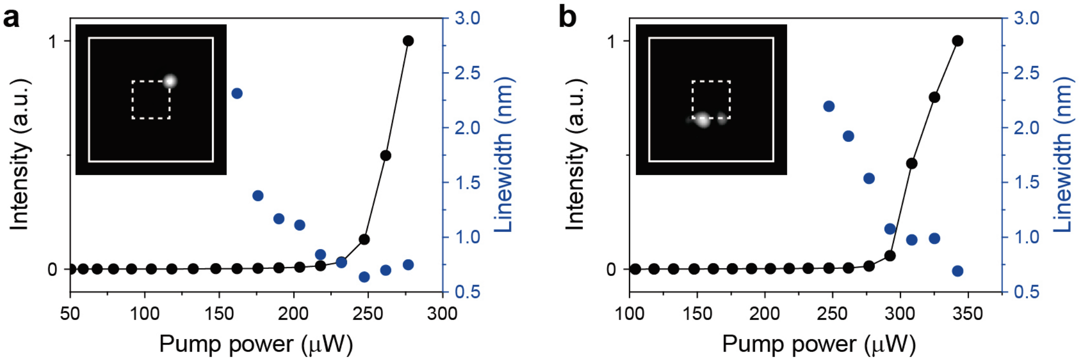

Supplementary Figure 9. Measurements of L-L curves and linewidth narrowing. Representative L-L curves and linewidth narrowing are shown for (**a**) corner-state and (**b**) edge-state lasing modes in Figs. 4b and c, respectively. Inset, measured mode images of Figs. 4b and c.

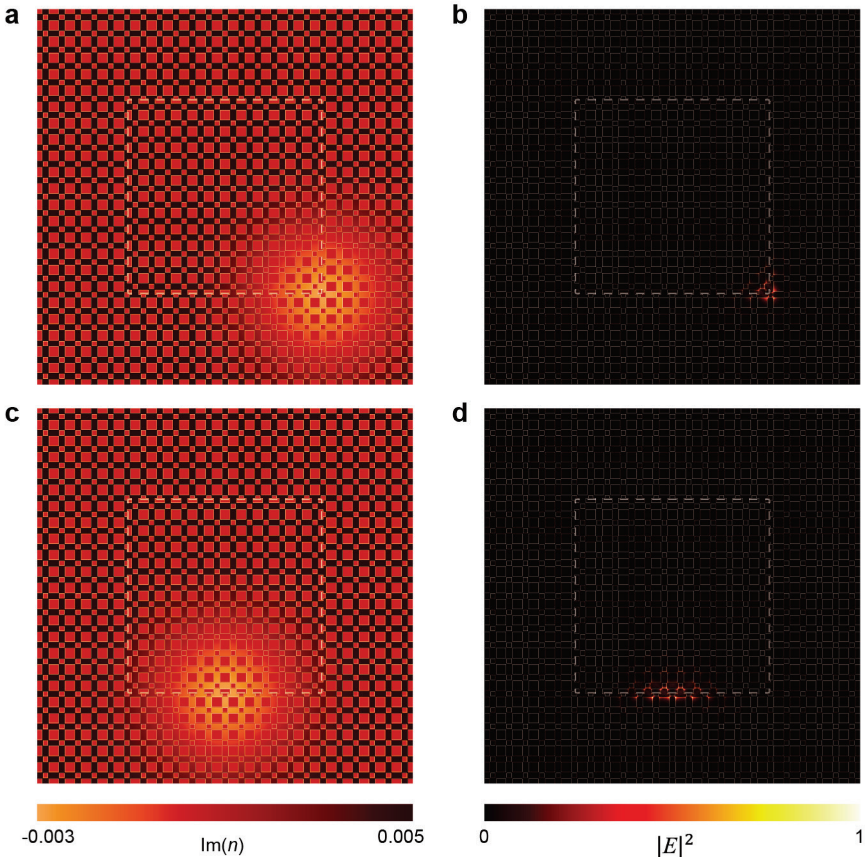

Supplementary Figure 10. Simulation of pump position-dependent emission profiles. A local optical gain is introduced to simulate the optical pumping at a corner (**a-b**) or an edge (**c-d**) in the PTI cavity with 12 × 12 topological unit cells. The structural parameters based on the SEM image of Figure 4a are used for FEM simulation: a = 500 nm, l_1 = 0.62 a, l_2 = 0.31 a, and h = 275 nm. **a** and **c,** Distributions of the imaginary part of the refractive index, Im(n). **b** and **d,** Calculated electric-field intensity ($|\mathbf{E}|^2$) profiles with frequencies of 207.1 THz (**b**) and 199.6 THz (**d**). These frequencies correspond to the wavelengths of 1448 nm (corner) and 1502 nm (edge), which agree well with the measured values of Figure 4d. The calculated Q factors of the corner (**b**) and edge (**d**) modes are 1.0×10^3 and 1.3×10^3, respectively, which significantly depend on the gain/loss contrast in the simulation. The white dashed squares indicate the interfaces between the trivial and nontrivial domains.

Supplementary Note 1

1. Tight-binding-model Hamiltonian

The tight-binding model of 2D SSH lattice including the next-nearest neighbour (NNN) hoppings, as shown in Supplementary Note Figure 1, under the Fourier transformation gives rise to the eigenvalue problem in the momentum space

$$\omega \boldsymbol{\psi} = \hat{H}(\mathbf{k})\boldsymbol{\psi} \tag{1}$$

with the Bloch Hamiltonian $\hat{H}(\mathbf{k}) = \hat{H}_{\mathrm{SSH}}(\mathbf{k}) + \hat{H}_{\mathrm{NNN}}(\mathbf{k})$ and wavefunction $\boldsymbol{\psi} = (B_1, A_2, B_3, A_4)^T$ being a four-component wavevector in the sublattice basis. In Supplementary Note Figure 1, two sublattices are labeled with subspaces (B, A), and the lattice period is set $a = 1$. Here, $\hat{H}_{\mathrm{SSH}}$ is the 2D generalization of the SSH model, which describes a dimerized chain with alternating couplings $t_{1,2}$ between nearest-neighbour (NN) sites, and $\hat{H}_{\mathrm{NNN}}(\mathbf{k})$ incorporates the NNN coupling terms. These two summands read

$$\hat{H}_{\mathrm{SSH}}(\mathbf{k}) = -t_1 \begin{pmatrix} 0 & 1 & 0 & 1 \\ 1 & 0 & 1 & 0 \\ 0 & 1 & 0 & 1 \\ 1 & 0 & 1 & 0 \end{pmatrix} - t_2 \begin{pmatrix} 0 & e^{-ik_x} & 0 & e^{-ik_y} \\ e^{ik_x} & 0 & e^{-ik_y} & 0 \\ 0 & e^{ik_y} & 0 & e^{ik_x} \\ e^{ik_y} & 0 & e^{-ik_x} & 0 \end{pmatrix}, \tag{2}$$

$$\hat{H}_{\mathrm{NNN}}(\mathbf{k}) = -\begin{pmatrix} d_{\mathrm{B}}(\mathbf{k}) & 0 & z_{\mathrm{B}}(\mathbf{k}) & 0 \\ 0 & d_{\mathrm{A}}(\mathbf{k}) & 0 & z_{\mathrm{A}}(\mathbf{k}) \\ z_{\mathrm{B}}^*(\mathbf{k}) & 0 & d_{\mathrm{B}}(\mathbf{k}) & 0 \\ 0 & z_{\mathrm{A}}^*(\mathbf{k}) & 0 & d_{\mathrm{A}}(\mathbf{k}) \end{pmatrix}, \tag{3}$$

where $d_{\mathrm{A,B}}(\mathbf{k}) = 2t'_{\mathrm{A,B}}(\cos k_x + \cos k_y)$, $z_{\mathrm{B}}(\mathbf{k}) = t_{\mathrm{B1}} + t_{\mathrm{B2}}e^{-ik_x} + t_{\mathrm{B4}}e^{-ik_y} + t_{\mathrm{B3}}e^{-i(k_x+k_y)}$, $z_{\mathrm{A}}(\mathbf{k}) = t_{\mathrm{A1}} + t_{\mathrm{A2}}e^{ik_x} + t_{\mathrm{A4}}e^{-ik_y} + t_{\mathrm{A3}}e^{i(k_x-k_y)}$. For our geometry, the hopping parameters relate as $t_{\mathrm{A1}} = t_{\mathrm{B1}}$, $t_{\mathrm{A3}} = t_{\mathrm{B3}}$, $t_{\mathrm{B2}} = t_{\mathrm{A4}} = t_{\mathrm{A2}} = t_{\mathrm{B4}}$, $t'_{\mathrm{A}} = t'_{\mathrm{B}}$. We assume a distance-decaying character of electromagnetic interactions. In the quasi-static approximation, the coupling coefficients are real-valued.

We first review the model with only NN couplings included and rewrite $\hat{H}_{\mathrm{SSH}}$ in the reordered basis $\boldsymbol{\psi} = (B_1, B_3, A_2, A_4)^T$ in the block form:

$$\hat{H}_{\mathrm{SSH}}(\mathbf{k}) = \begin{pmatrix} 0 & \hat{Q} \\ \hat{Q}^{\dagger} & 0 \end{pmatrix}, \tag{4}$$

$$\hat{Q} = -\begin{pmatrix} t_1 + t_2 e^{-ik_x} & t_1 + t_2 e^{-ik_y} \\ t_1 + t_2 e^{ik_y} & t_1 + t_2 e^{ik_x} \end{pmatrix}, \tag{5}$$

This Hamiltonian is off-diagonal and hence satisfies a chiral (sublattice) symmetry

$$\hat{\tau}_3 \hat{H}_{\mathrm{SSH}}(\mathbf{k}) \hat{\tau}_3^{\dagger} = -\hat{H}_{\mathrm{SSH}}(\mathbf{k}), \tag{6}$$

where the chiral operator $\hat{\tau}_3 = \hat{\sigma}_z \otimes \hat{\mathbb{I}}_{2\times 2}$ is the third Pauli matrix acting on the (B, A) subspace.

While the chiral symmetry of total Hamiltonian $\hat{H}$ is broken, for our geometry, it still respects C_{4v} symmetry, similar to the basic $\hat{H}_{\mathrm{SSH}}$ part, obeying the property $\hat{r}_4 \hat{H}(\mathbf{k}) \hat{r}_4^{\dagger} = \hat{H}(\hat{R}_4 \mathbf{k})$, where the rotation operator $\hat{r}_4$ is given by

$$\hat{r}_4 = \begin{pmatrix} 0 & 0 & 0 & 1 \\ 1 & 0 & 0 & 0 \\ 0 & 1 & 0 & 0 \\ 0 & 0 & 1 & 0 \end{pmatrix}, \tag{7}$$

and matrix $\hat{R}_4$ rotates the momentum by angle $\pi/2$: $\hat{R}_4(k_x, k_y) = (-k_y, k_x)$. The presence of C_{4v} symmetry in the lattice allows distinguishing two topological phases, depending on the ratio of inter- and intra- cellular spacing. The topological crystalline phase is encoded by the parities of eigenfunctions at high-symmetry points of the Brillouin zone (see e.g. Supplementary Figure 2). Simultaneous presence of C_{4v} and chiral symmetries in a standard 2D SSH model, with the NN hopping only, pins the frequency of corner states to the zero-energy level in the middle of the spectrum. In this way, corner modes appear embedded within the bulk bands of the lattice (see Supplementary Note Figure 2c). However, to avoid mode competition in the lasing process, it is desirable to spectrally isolate corner states. Broken chiral symmetry and local boundary effects modify the band structure, so that corner modes fall inside the bandgap (see Supplementary Note Figure 2d and e). For illustrative purposes, it is sufficient to retain only dominating NNN bonds, which correspond to the diagonal intra-cellular coupling $t_{\mathrm{A1}} = t_{\mathrm{B1}}$ of the sites in the shrunken quadrumers' domain ($|t_1| > |t_2|$), and transform to the inter-cellular coupling $t_{\mathrm{A3}} = t_{\mathrm{B3}}$ in the expanded quadrumers' domain ($|t_1| < |t_2|$).

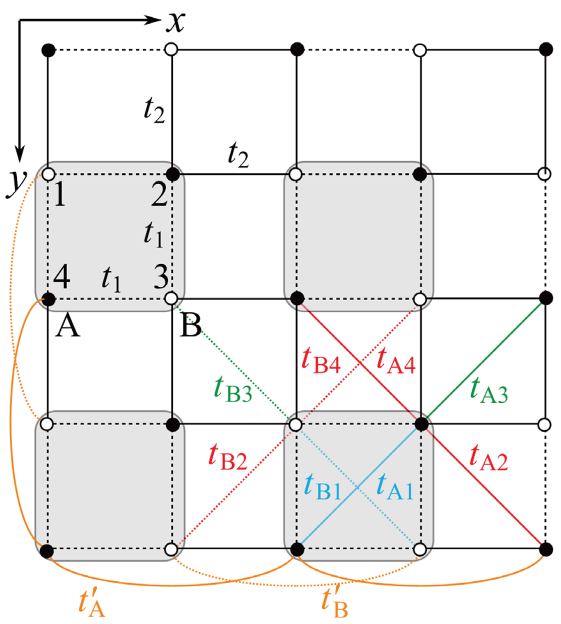

Supplementary Note Figure 1. Schematic of a square 2D SSH lattice with NN and NNN hopping. Four sites in each unit cell compose quadrumers framed in gray color.

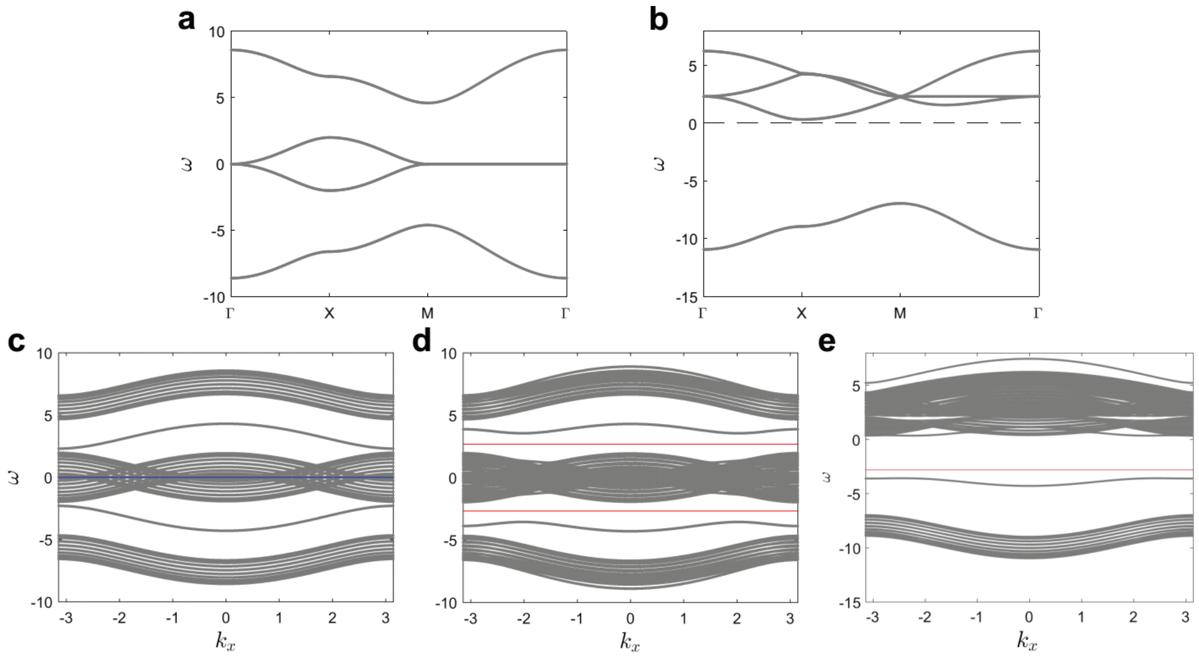

Supplementary Note Figure 2. Illustration of typical TBM spectra for 2D SSH lattices. Top row: Bulk band structures along high-symmetry directions in the 2D SSH lattice with (**a**) NN coupling only, (**b**) both NN and NNN coupling included. The dashed line in (**b**) depicts a sum of the eigenfrequencies $\sum_{i=1}^{4} \omega_i(\mathbf{k}) = 0$ over four bands. Bottom row: Band diagrams for supercells

made of (**c**) expanded quadrumers without NNN interactions and open boundary conditions (blue line marks the zero-energy corner-state frequency); (**d**) stitched shrunken and expanded domains without NNN interactions and strong bond $t_w = 2.8$ at the interface (reddish lines mark the corner state frequencies falling into the bandgaps); (**e**) the same as (**d**) but with NNN interactions added (superimposed reddish line marks the corner state frequency within the band gap). Parameters in the shrunken domain: $t_1 = 3.3$, $t_2 = 1$, $t_{\mathrm{A1}} = t_{\mathrm{B1}} = t_1/\sqrt{2}$; in the expanded domain: $t_1 = 1$, $t_2 = 3.3$, $t_{\mathrm{A3}} = t_{\mathrm{B3}} = t_2/\sqrt{2}$. The introduced domain-wall defect only alters the edge and corner state frequencies. NNN interactions break a chiral symmetry making the spectrum asymmetric but do not eliminate edge and corner modes.

2. Corner states

We first obtain an analytical solution for corner states in a lattice of expanded quadrumers without NNN interactions. We solve TBM equations for the real-space eigenfunction of our system indexing each quadrumer by a pair of integer coordinates (x, y):

$$\omega\psi_{B_1}(x,y) = -t_1(\psi_{A_2}(x,y) + \psi_{A_4}(x,y)) - t_2\,(\psi_{A_2}(x-1,y) + \psi_{A_4}(x,y-1)),$$

$$\omega\psi_{A_2}(x,y) = -t_1(\psi_{B_1}(x,y) + \psi_{B_3}(x,y)) - t_2\,(\psi_{B_1}(x+1,y) + \psi_{B_3}(x,y-1)),$$

$$\omega\psi_{B_3}(x,y) = -t_1(\psi_{A_2}(x,y) + \psi_{A_4}(x,y)) - t_2\,(\psi_{A_2}(x,y+1) + \psi_{A_4}(x+1,y)),$$

$$\omega\psi_{A_4}(x,y) = -t_1(\psi_{B_1}(x,y) + \psi_{B_3}(x,y)) - t_2\,(\psi_{B_1}(x,y+1) + \psi_{B_3}(x-1,y)).$$

Given the chiral symmetry, we look for the zero-energy corner state at $\omega = 0$ focusing on the corner located at $x = y = 1$ in a semi-infinite structure with x and y ranging from 1 to infinity. The open boundary condition is specified by $\psi_{B_1,B_3,A_2,A_4}(0,0) = 0$, $\psi_{B_1,B_3,A_2,A_4}(x,0) = 0$, $\psi_{B_1,B_3,A_2,A_4}(0,y) = 0$. We also require the wavefunction decay at infinity in both x and y directions. Applying the boundary conditions, we find the corner state solution

$$\psi_{B_1}(x,y) = \left(-\frac{t_1}{t_2}\right)^{x-1}\left(-\frac{t_1}{t_2}\right)^{y-1}, \tag{8}$$

$$\psi_{A_2}(x,y) = \psi_{A_4}(x,y) = \psi_{B_3}(x,y) = 0,$$

which has non-zero amplitudes only on B_1 sublattice, and decays away from the corner provided $|t_1| < |t_2|$.

Next, we activate NNN interactions and examine the associated modifications. Considering the diagonal terms proportional to $t'_{\mathrm{A,B}}$ in Supplementary Equation 3 vanishing, we notice that the Hamiltonian $\hat{H}_1 \equiv \hat{H}$ satisfies the generalized chiral symmetry defined through the following transformations

$$\begin{aligned} &\Gamma_4^{-1}\hat{H}_1\Gamma_4 = \hat{H}_2, \\ &\Gamma_4^{-1}\hat{H}_2\Gamma_4 = \hat{H}_3, \\ &\Gamma_4^{-1}\hat{H}_3\Gamma_4 = \hat{H}_4, \\ &\hat{H}_1 + \hat{H}_2 + \hat{H}_3 + \hat{H}_4 = 0, \end{aligned} \tag{9}$$

with operator Γ_4, being a diagonal matrix so that $\Gamma_4^4 = \hat{\mathbb{I}}_{4\times4}$:

$$\Gamma_4 = \begin{pmatrix} 1 & 0 & 0 & 0 \\ 0 & e^{\frac{i\pi}{2}} & 0 & 0 \\ 0 & 0 & e^{i\pi} & 0 \\ 0 & 0 & 0 & e^{\frac{i3\pi}{2}} \end{pmatrix}. \tag{10}$$

The Hamiltonians $\hat{H}_{1,2,3,4}$ have the same set of four eigenvalues $\omega_{1,2,3,4}$. By taking the trace of the fourth line in Supplementary Equation 9, we obtain the equality

$$\mathrm{Tr}(\hat{H}_1 + \hat{H}_2 + \hat{H}_3 + \hat{H}_4) = 4\mathrm{Tr}(\hat{H}_1) = 0, \tag{11}$$

which ensures the zero sum of four eigenvalues $\sum_{i=1}^{4} \omega_i = 0$, as shown in Supplementary Note Figure 2b. Such a generalized chiral symmetry is behind the improved stability of corner states compared to the non-topological defect states, as also supported by TBM calculations in Supplementary Note Figure 3. This property assumes the model Hamiltonian may only be perturbed by hopping processes.

Omitting the NNN coupling, the system restores to the chiral-symmetric. To tackle the eigenvalue problem $\hat{H}_1\psi = \omega\psi$, we employ a perturbative approach with the total Hamiltonian decomposed into two parts $\hat{H}_1 = \hat{H}_1^{(0)} + \hat{H}_1^{(1)} = \hat{H}_{\mathrm{SSH}} + \hat{H}_{\mathrm{NNN}}$, implying that both the parts possess the property (Supplementary Equation 9). Assume the eigenstate $\psi_j^{(0)}$ of the zero-order Hamiltonian, $\hat{H}_1^{(0)}\psi_j^{(0)} = \omega^{(0)}\psi_j^{(0)}$, resides on only one sublattice B_1, A_2, B_3, A_4 with subscript $j = 1,2,3,4$, respectively. Under the action of Γ_4, $\psi_j^{(0)}$ transforms as

$$\Gamma_4\psi_j^{(0)} = e^{\frac{i(j-1)\pi}{2}}\psi_j^{(0)}. \tag{12}$$

Hence, from Supplementary Equations 9 and 12, we obtain

$$(\hat{H}_1^{(0)}+\hat{H}_2^{(0)}+\hat{H}_3^{(0)}+\hat{H}_4^{(0)})\psi_j^{(0)}=\sum_{i=1}^{4}\Gamma_4^{-(i-1)}\hat{H}_1^{(0)}\Gamma_4^{i-1}\psi_j^{(0)}=4\omega^{(0)}=0, \quad (13)$$

stipulating $\omega^{(0)}=0$ for any mode that occupies only one sublattice, which is the corner state (Supplementary Equation 8). We look for the corrections to the eigenstate $\psi_j \approx \psi_j^{(0)}+\Delta\psi_j^{(1)}+\Delta\psi_j^{(2)}$ and eigenfrequency $\omega \approx \omega^{(0)}+\omega^{(1)}+\omega^{(2)}$, where $\omega^{(0)}=0$ and $\hat{H}_1^{(0)}\psi_j^{(0)}=0$. Since $\langle\psi_j^{(0)}|\hat{H}_{\mathrm{NNN}}|\psi_j^{(0)}\rangle=0$ due to Supplementary Equation 9, the first-order correction $\omega^{(1)}$ vanishes, and the second-order correction to the corner state frequency is expressed as $\omega^{(2)}=\frac{\langle\psi_j^{(0)}|\hat{H}_{\mathrm{NNN}}\left(\hat{H}_1^{(0)}\right)^{-1}\hat{H}_{\mathrm{NNN}}|\psi_j^{(0)}\rangle}{\langle\psi_j^{(0)}|\psi_j^{(0)}\rangle}$, which suggests that the frequency only slightly deviates from the zero level for the dimerization contrast $|t_2/t_1|>3$.

Together with experimental data and numerical tests of robustness for a realistic system performed in full-wave modelling, which show that corner modes remain well isolated spectrally from bulk modes upon introducing disorder and imperfections, the above complementary TBM analysis confirms sustainability of the corner states in our structure for practical purposes.

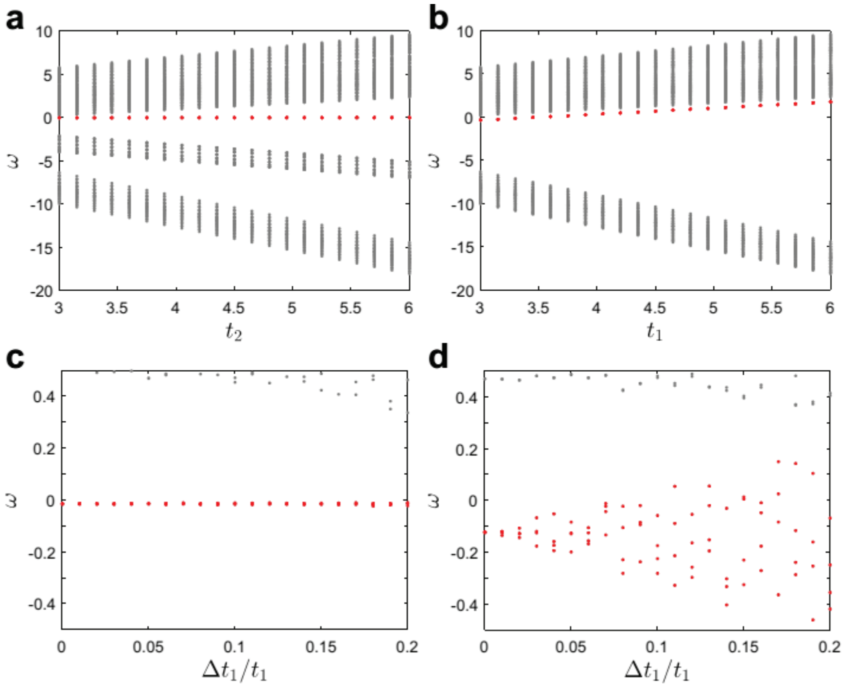

Supplementary Note Figure 3. Stability of corner modes in finite discrete lattices. a,b Dependence of eigenfrequencies on the strong coupling strength in finite lattices made of 7 by 7

(**a**) expanded quadrumers with $t_1 = 1$, $t_{\mathrm{A}3} = t_{\mathrm{B}3} = t_2/\sqrt{2}$; (**b**) shrunken quadrumers with $t_2 = 1$, $t_{\mathrm{A}1} = t_{\mathrm{B}1} = t_1/\sqrt{2}$. The non-topological defect states in (**b**) are created by the frequency detuning $\Delta\omega = M$ with a mass parameter $M = -4$ at the four utmost corner sites of the lattice. Red points mark the modes localized at the corners, and gray dots correspond to bulk modes. **c**,**d** Eigenfrequencies in the zoomed-in spectrum range near $\omega = 0$ when the disorder over the (**a**,**b**) lattices is introduced into the intra-quadrumer tunneling amplitude t_1. Δt_1 denotes the standard deviation for the tunneling strength t_1. The introduced perturbations respect the four-fold rotation and generalized chiral symmetries of the unit cells. Calculations are performed for (**c**) $t_1 = 1$, $t_2 = 3.3$, $t_{\mathrm{A}3} = t_{\mathrm{B}3} = t_2/\sqrt{2}$; (**d**) $t_1 = 3.3$, $t_2 = 1$, $t_{\mathrm{A}1} = t_{\mathrm{B}1} = t_1/\sqrt{2}$. The frequencies of non-topological defect states in (**b**,**d**) undergo significantly larger deviations, compared to those of the corner states in (**a**,**c**).
